# Evaluation of single-layer versus double-layer suturing of low transverse uterine incisions in cesarean section and follow-up of scars by ultrasound: a prospective randomized controlled study

**DOI:** 10.55730/1300-0144.5906

**Published:** 2024-11-05

**Authors:** Erhan DEMİRDAĞ, Hazal KUTLUCAN, Anıl Doğukan TUTAL, Bilge Pınar ÇALIŞKAN KESKİNSOY, Gülşah KARAKUYU, Recep Onur KARABACAK

**Affiliations:** 1 Department of Obstetrics and Gynecology, Faculty of Medicine, Gazi University, Ankara, Turkiye; 2 Department of Obstetrics and Gynecology, Faculty of Medicine, TOBB Economy and Technology University, Ankara, Turkiye

**Keywords:** Cesarean section, scar, niche, suture techniques, ultrasound

## Abstract

**Background/aim:**

Cesarean section (CS) is a widely performed operation worldwide but data about uterine closure are lacking. We aimed to evaluate scar niches and compare single-layer and double-layer uterine closure at 6 months following CS.

**Materials and methods:**

This prospective randomized trial assessed 56 women undergoing single- or double-layer uterine closure. None of the patients had previous uterine surgery and all CS cases were elective. Transvaginal ultrasound was performed 6 months after CS to assess the uterine scars by measuring the width, depth, and length of the scar niche and residual myometrial thickness. An experienced sonographer was blinded to the uterine closure technique and the ultrasounds were conducted by practitioners unaware of the technique in the postoperative follow-up appointments.

**Results:**

Twenty-eight women were assigned to the single-layer closure group (Group 1) and 28 were assigned to the double-layer closure group (Group 2). The demographic and clinical characteristics of patients and the width, depth and diameter of the niche were similar between the groups, as was residual myometrial thickness. There was no difference in uterine scar volume under the incision between the two groups. The duration of surgery was approximately 5 min longer (p = 0.048) and hemoglobin decrease was about 0.5 g/dL less (p = 0.039) in the double-layer group compared to the single-layer group. Postmenstrual spotting rates were similar between the groups. Group 1 had two and Group 2 had one spontaneous pregnancy within 6 months after CS.

**Conclusion:**

The single- and double-layer closure techniques do not produce different impacts on CS niche features at 6 months after delivery. Ultrasound might be an important noninvasive diagnostic tool for understanding CS scar remodeling.

## 1. Introduction

Cesarean section (CS) is the most common operation in obstetrics and gynecology worldwide [1]. The consistently rising rate of CS procedures has become a significant concern [1], and related complications such as isthmocele have generated interest in focusing on suture techniques and CS scar-related potential morbidities [2].

Growing attention to the issue of scar healing has led researchers to investigate optimal uterine closure techniques. Studies comparing these techniques have demonstrated various advantages and disadvantages. It is generally reported that single-layer closure provides shorter operation times, decreased blood loss, and shorter hospitalization periods [3], although incomplete closure of the uterine wall is more likely to be seen with single-layer procedures [4], which may contribute to isthmocele formation. In addition, there is still no consensus about the relation between closure techniques and myometrial thickness in the literature. It was indicated that single-layer interlocking closure is associated with thinner myometrial thickness [5,6]. However, some researchers found that double-layer closure did not increase myometrial thickness as measured a few months after delivery [7]. Furthermore, the number of uterine ruptures that might reflect myometrial thickness was higher by one patient following double-layer closure in the CORONIS trial with a large sample size [8]. Thus, despite many comparative studies having been conducted to date, the optimal uterine incision suturing technique still needs to be clarified.

Postpartum uterine scar evaluation generally begins with ultrasound guidance, which is a validated method for assessing uterine scar defects [9,10]. The visualization of scars using ultrasound might be advantageous, although appropriate uterine scar thickness values in the context of single-versus double-layer closure are not fully understood [11]. Furthermore, there is limited evidence for the evaluation of defects based on scar appearance [2]. Thus, it is necessary to clarify the value of one technique over the other, which has not yet been clearly defined.

In light of these gaps in the literature, we aimed to identify the optimal uterine closure technique by comparing measurements of the cesarean scar niche, including the width, depth, and diameter of the niche and residual myometrial thickness (RMT), at 6 months after CS procedures. We also aimed to evaluate the scar volume of the niche after these techniques via transvaginal ultrasound (TVUS) in a randomized prospective setting.

## 2. Materials and methods

**Figure 1:**
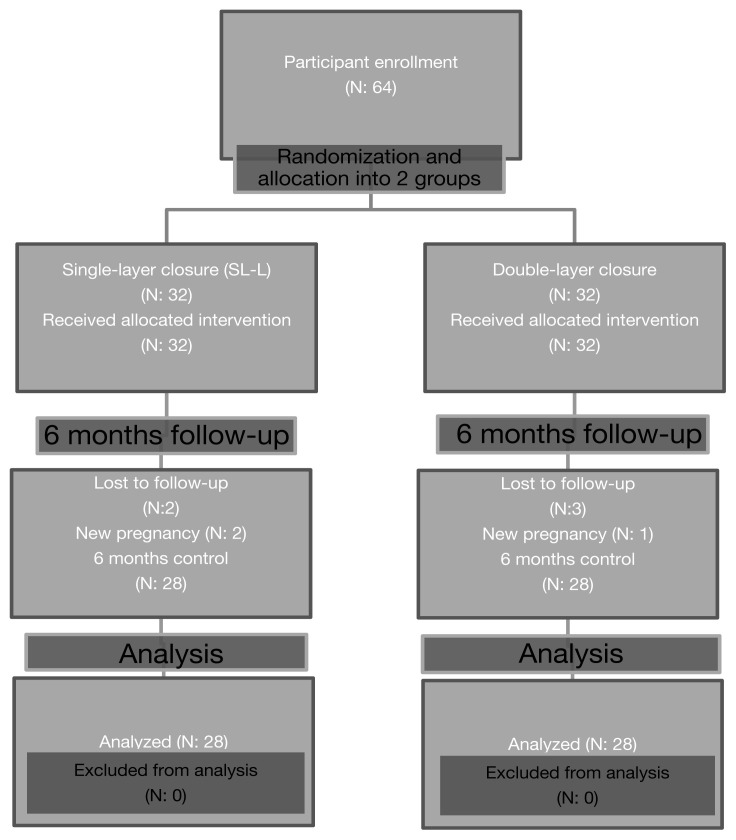
Study flowchart.

This prospective randomized study was conducted in the Gazi University Faculty of Medicine Hospital between December 2018 and October 2021 with 64 postpartum patients. The study was approved by the Gazi University Clinical Research Ethics Committee, confirming its conformity with the Declaration of Helsinki (Approval No. 2018/237), and it was registered at clinicaltrials.gov on September 18, 2018 (No. NCT03676907). Patients who were willing to participate in the study with informed consent, who were undergoing planned elective CS between 37^0/7^ and 40^6/7^ weeks of gestation without emergent conditions, and who had term singleton pregnancies were included in the study. The week of pregnancy was confirmed based on the last menstrual period and first trimester crown–rump length as measured by ultrasonography. Patients with multiple pregnancies, fetal presentation anomalies, suspected macrosomia, placental abnormalities, previous uterine surgery, uterine malformation, infectious or connective tissue disorders or diabetes mellitus, CS conducted under emergent conditions, a need for at least two additional sutures during uterine closure, or newly diagnosed pregnancy in the 6-month follow-up period were excluded. We also excluded patients who went into labor before CS to eliminate the effect of labor on the lower uterine segment. Because of these strict inclusion criteria, all included cases entailed maternal requests for elective CS. The flowchart of the study is presented in Figure 1.

CS was performed under regional anesthesia by the same surgeon (E.D.) for all patients included in this study. After Pfannenstiel incision, the vesicouterine fold was separated by blunt and sharp dissection and a lower transverse uterine segment incision was performed in all procedures. After delivery of the fetus and placenta, the incision was closed with single- or double-layer suturing using multifilament synthetic braided absorbable polyglactin suture material (60–90 days; Vicryl 1.0, Ethicon, Cincinnati, OH, USA). The single-layer technique with locking sutures (SL-L) was performed by suturing approximately 1 cm of tissue from the upper and lower segments; the mucosa and muscular layer were stitched together and locked continuously with approximately 1-cm intervals. The double-layer suture technique was carried out in two stages. In the first stage, the endometrium was closed by taking about 0.5 cm of tissue from the lower and upper segments with continuous locking suturing at intervals of approximately 1 cm, similar to SL-L method. In the second stage, the muscular layer was taken approximately 1 cm from the lower and upper segments, and continuous (lockless) suturing with intervals of about 1 cm was carried out. The double layer was constructed by the first layer of locking and the second layer by continuous suturing; this was referred to as the double-layer technique with locking and continuous sutures (DL-LC). Patients were excluded if there was a need for at least two additional sutures in the course of the operation due to the possible effects on surgical outcomes and scar niche characteristics. The operation was completed after confirming hemostasis. Prophylactic antibiotic treatment (2 g of intravenous cephazolin sodium) and 20 IU of oxytocin in intravenous fluid were administered during the CS procedure for all patients. The operation time was recorded by operating room technicians.

Participants were allocated into two groups. Randomization was performed according to unique patient identification numbers assigned automatically with the computer-based hospital system at the time of first admission. In the operating room, the nurse informed the surgeon about the patient’s identification number before surgery. Patients with numbers ending in odd digits (1, 3, 5, 7, 9) were included in the SL-L group (Group 1) and those with numbers ending in even digits (0, 2, 4, 6, 8) were included in the DL-LC group (Group 2). The suture technique, duration of surgery, and observed blood loss were noted. In addition, patients’ demographic features, duration of hospitalization, infant birth weight, and hemoglobin level changes in the first postoperative 24 h were recorded. Blood loss was measured with a suction bottle, which was initially reset before surgery.

**Figure 2:**
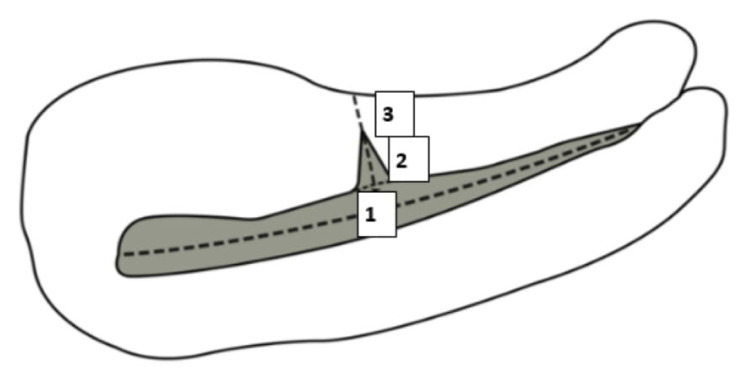
Schematic diagram of the uterus in the sagittal plane with demonstration of the two dimensions, the width of the defect (1), the depth of the defect (2), and residual myometrial thickness (3).

**Figure 3:**
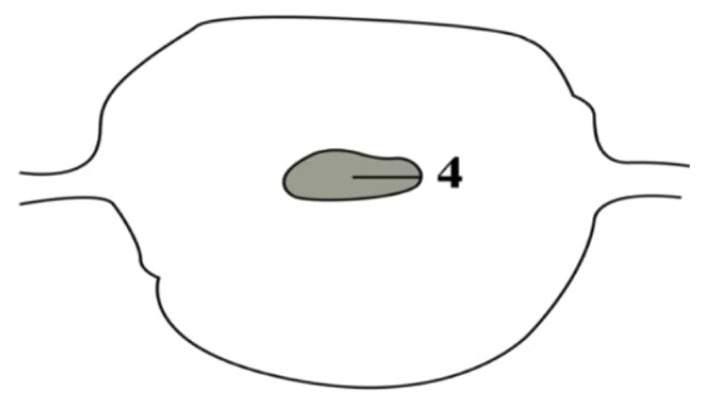
Schematic diagram of the uterus in the axial plane with the diameter of the defect (4).

Patients were examined again 6 months after surgery in the supine position with an empty bladder via TVUS (Voluson E6, GE Healthcare, Tiefenbach, Austria) by the same obstetrician (A.D.T.) regardless of menstrual phase; this obstetrician had not participated in the previous operations. The participants and the experienced sonographer obstetrician were blinded to the closure technique. Measurements were conducted while the endometrium, lower uterine segment, and cervix were being visualized in the sagittal plane. On this plane, a wedge-shaped hypoechoic image causing discontinuity in the structure of the endometrium and disconnection from the front line towards the serosa was defined as a scar defect. The width and depth of the defect in the sagittal plane and its length in the axial plane were measured (Figures 2 and 3).

**Figure 4:**
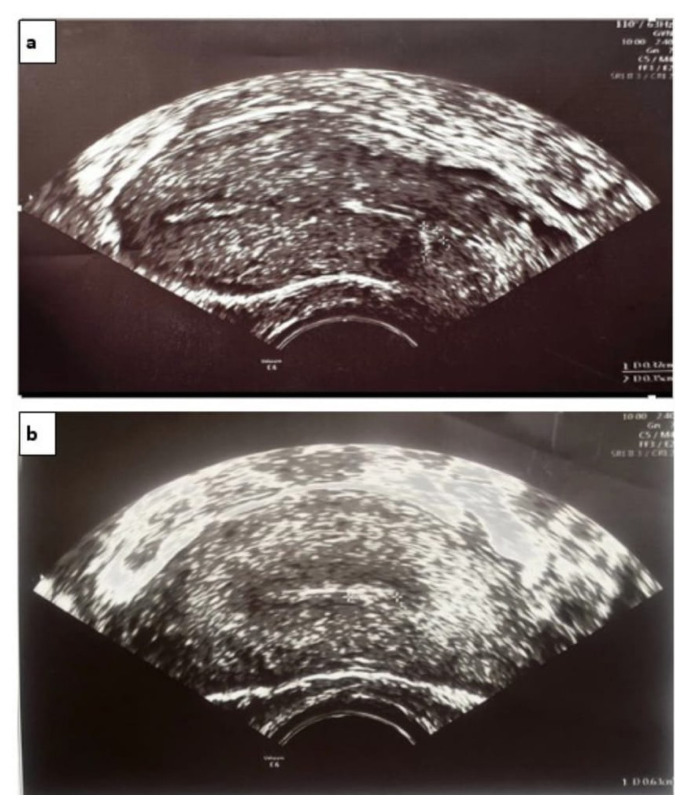
Transvaginal ultrasonographic images portraying uterine scar niche measurements at 6 months after surgery. Measurement of the width and height of the defect and appearance of the residual myometrial thickness (a); diameter of the defect (b).

With these measurements, volumetric data were obtained sonographically, multiplying the width and depth of the defect in the sagittal plane and the axial length by π × 4/3 as stated by Raine-Fenning et al. [12]. The length of the intact myometrial tissue extending vertically from the lower edge of the scar defect to the serosa (i.e., the distance between the uterine serosa and outer edge of the cesarean scar) was measured as RMT (Figure 2). The width, depth, and length of the uterine scar niche and RMT were measured by TVUS at 6 months after the surgery (Figure 4a and 4b).

All patients were in the breastfeeding period and were not using any contraceptive methods, including intrauterine devices or hormonal drugs, during the follow-up period.

Based on a metaanalysis [13], uterine scar defects were defined as defects of approximately 25% with single-layer sutures and approximately 61% with double-layer sutures. Considering a statistical significance threshold of p < 0.05 and study power of 0.80, the necessary sample size was calculated as a minimum of 28 for each group.

The primary outcome measures in this study were echo criteria including uterine scar defect volume (SDV), RMT, niche prevalence, and the width, depth, and length of the scar defect. The secondary outcome measures were duration of CS, postmenstrual spotting, and dysmenorrhea rates at 6 months after surgery. The duration of CS was taken as the interval between the abdominal incision and the end of skin closure. Postmenstrual spotting was defined as small amounts of vaginal bleeding after normal menstrual bleeding ceased. Postmenstrual spotting and dysmenorrhea were evaluated as the most commonly encountered complaints after CS due to isthmocele.

Data were analyzed using IBM SPSS Statistics 21.0 (IBM Corp., Armonk, NY, USA). Variables of interest were evaluated for normal distribution using analytical (skewness and kurtosis, Kolmogorov–Smirnov test) and visual (histograms, probability plots) methods. Student’s t-test and the Mann–Whitney U test were used for normally distributed and nonnormally distributed metric data, respectively. Categorical variables were compared by chi-square test or Fisher’s exact test. Data were presented as mean ± standard deviation (SD), median (25th–75th percentile), and percentage values. Statistical significance was accepted at p < 0.05.

## 3. Results

A total of 64 women were included in this study. TVUS was not performed for 8 patients due to new pregnancy (n = 3, 4.7%) or the patient being lost to follow-up (n = 5, 7.8%). As a result, 28 patients underwent CS with the SL-L technique (Group 1) and 28 patients underwent CS with the DL-LC technique (Group 2). None of the patients had undergone CS or any other uterine surgery before and all of them were in the term gestational period at the time of CS.

The demographic and clinical characteristics of the groups are shown in Table 1. The mean age, body mass index, gestational age at delivery, birth weight, and nulliparity rates of the patients were comparable between the two groups. Comparisons of surgical and clinical outcomes between the groups are presented in Table 2. Niche rates, median RMT and SDV, and the mean width, depth, and diameter of the scar defect were similar between the groups. Clinical symptoms including postmenstrual spotting and dysmenorrhea at 6 months after surgery were similarly not significantly different between the groups. However, the mean duration of surgery was significantly longer in Group 2 (46 ± 9 min) compared to Group 1 (41 ± 9 min) (p = 0.048). The mean change in hemoglobin level was significantly lower in Group 2 (1.5 ± 1 g/dL) than in Group 1 (2 ± 0.9 g/dL) (p = 0.039).

## 4. Discussion

This prospective randomized study assessed the relationship between uterine closure techniques and CS scar defect features. We found that niche prevalence, RMT, SDV, postmenstrual spotting, and dysmenorrhea rates were comparable between the single- and double-layer uterine closure techniques. However, the duration of CS was significantly longer and the change in hemoglobin level at the 24th postoperative hour was significantly lower with the double-layer closure technique.

There is still no consensus about the best uterine closure technique in terms of incomplete healing following CS. Some previous studies have found an association between uterine scar healing and various gynecological problems and obstetric outcomes in further pregnancies [3,14,15]. While incomplete healing of the scar may be associated with scar morbidities such as dehiscence in pregnancy, placental invasion anomalies, and uterine rupture, there is still no evidence to clarify the best technique.

Despite improvements in imaging methods, the gold standard approach for evaluating uterine scars is controversial in the literature. Kataoka et al. [16] and Bamberg et al. [6] assessed the uterine niche and residual myometrium via sonographic middle-sagittal view and saline contrast sonography. Another study compared ultrasonography and hysteroscopy [17]. Although there are different opinions on assessing the uterine niche, we used noninvasive TVUS to evaluate uterine incisions.

There have been many attempts to describe uterine scars by ultrasound, but methods for assessing the integrity of these scars are not yet supported by evidence [3]. TVUS was found to be more reliable than transabdominal ultrasound for assessing the lower uterine segment [18,19]. In contrast, we evaluated the SDV using a specified formula to describe uterine scars and we did not find any difference between the groups. According to a previous study, the defect of the uterine scar was related to not only the suturing technique but also the closure of the cervix and proper drainage of the uterine cavity [11]. For this reason, we only included elective CS cases in our study to exclude secondary contributing factors like cervical dilatation, duration of labor, or oxytocin augmentation.

Generally, the medical literature does not specify a precise puerperal evaluation time for uterine scar recovery or the superiority of single- or double-layer closure regarding scar defect configuration and complications. One prospective study concluded that myometrial scar tissue recovers within 6 months based on evaluations by magnetic resonance imaging [20]. In another randomized study, patients were followed for 2 years, although the first control was performed 6 weeks after surgery, and it was found that RMT decreased significantly but remained stable after 6 months postpartum while scar thickness was significantly increased with the double-layer approach [14]. In another study, the defect length was greater after single-layer closure at 6 months after surgery [15]. In the present study, we screened for complete uterine involution to ensure sufficient time for the recovery of uterine anatomy and we identified the optimal control time as 6 months. RMT and defect length were similar between our study groups. These findings could be attributed to the homogeneous study population with the application of strict selection criteria. Some previous studies similarly concluded that single-layer closure was associated with a higher risk of dysmenorrhea than double-layer closure [7,8,21,22]. In a multicenter trial with a large sample size, no differences in median postmenstrual spotting days or dysmenorrhea scores were observed between the two arms [23]. In our study, although niche rates were relatively lower in the SL-L group and there was a trend toward more postmenstrual spotting and dysmenorrhea in this group, we did not find any statistically significant difference between the groups. These gynecological symptoms may have multiple causes other than isthmocele in the postpartum period, which may have contributed to our results. However, our findings may also be due to the relatively small sample size of the study.

Concerning isthmocele formation and infertility, our study revealed similar distributions of isthmic niche formation and new post-CS pregnancy between the groups. In a previous study, similar niche rates were also found between single- and double-layer closure groups [24]. These variables do not seem to be associated with the uterine closure technique itself.

The main strength of the current study lies in the prospective design and randomization of groups in a tertiary center. Other strengths include surgeries having been performed by the same surgeon, having used the same suture material and technique in both groups, and ultrasonography evaluations having been performed by a single experienced sonographer obstetrician blindly, which may have provided standardization. The major limitations of our study were the small sample size due to strict inclusion criteria and the fact that reaching the minimum sample size took a long time. However, we obtained significant results for the duration of surgery and hemoglobin level changes. Another limitation was that patients were evaluated on random days of their menstrual cycles, which may have adversely affected synchronization.

In conclusion, the CS rate is increasing dramatically worldwide and more women are being exposed to uterine scars as a result. Ultrasound is a noninvasive diagnostic approach for understanding scar remodeling and measuring SDV. This study showed that single- and double-layer closure techniques are not superior to each other in terms of niche rate, uterine scar measurements, SDV (isthmocele), postpartum dysmenorrhea, and vaginal spotting at 6 months. Our results may offer guidance to clinicians in deciding between these closure techniques during surgery and may suggest novel directions for further studies regarding myometrial wound healing.

## Figures and Tables

**Table 1:** Comparison of demographic and clinical characteristics between the groups.

Variable	Uterine closure technique		p-value
	SL-L group (n=28)	DL-LC group (n=28)	
Age, years	28.11 ± 5.9	27.32 ± 4.3	0.57
Nulliparity, n (%)	23 (82)	25 (89)	0.71
Body mass index, kg/m^2^	27.7 ± 4.7	29.4 ± 5.0	0.20
Gestational age at cesarean, weeks	38.8 ± 0.6	38.9 ± 0.9	0.60
Birthweight, gram	3553.9 ± 476.5	3299.3 ± 493.9	0.06

Data were presented as mean ± standard deviation (SD) and percentage. p < 0.05 was considered significant.

**Table 2:** Comparison of clinical outcomes between the SL-L and DL-LC groups.

Variable	Uterine closure technique		p-value
	SL-L group (n=28)	DL-LC group (n=28)	
Duration of surgery (minutes)	41 ± 9	46 ± 9	**0.048**
Hemoglobin level change (g/dL)	2 ± 0.9	1.5 ± 1	**0.039**
Observed blood loss at surgery (mL)	600 (447–750)	575 (450–737)	0.83
Duration of hospitalization (day)	1 (1–2)	1 (1–2)	0.91
Additional hemostatic suture, n	0.5 (0–1)	1 (0–1)	0.23
Niche prevalence, n (%)	21 (75)	23 (82.1)	0.52
Depth of niche (mm)	4.8 ± 1.6	4.5 ± 1.6	0.55
Width of niche (mm)	5.4 ± 1.5	5.2 ± 1.4	0.60
Diameter of niche (mm)	5.6 ± 1.2	5.3 ± 1.4	0.32
Residual myometrial thickness (mm)	4.55 (3.35–5.35)	5 (4.2–5.67)	0.12
Myometrial defect volume (mm^3^)	600 (447–750)	575 (450–737)	0.83
Postmenstrual spotting, n (%)	6 (21.4)	4 (14.3)	0.49
Dysmenorrhea, n (%)	7 (25)	2 (7)	0.14

Data were presented as mean ± standard deviation (SD), median (25–75 percentile), and percentage. p < 0.05 was considered significant.
